# Toxicity of Large and Small Surface-Engineered Upconverting Nanoparticles for In Vitro and In Vivo Bioapplications

**DOI:** 10.3390/ijms25105294

**Published:** 2024-05-13

**Authors:** Lucia Machová Urdzíková, Dana Mareková, Taras Vasylyshyn, Petr Matouš, Vitalii Patsula, Viktoriia Oleksa, Oleksandr Shapoval, Magda Vosmanská, David Liebl, Aleš Benda, Vít Herynek, Daniel Horák, Pavla Jendelová

**Affiliations:** 1 Institute of Experimental Medicine, Czech Academy of Sciences, Vídeňská 1083, 14220 Prague, Czech Republic; lucia.machova@iem.cas.cz (L.M.U.); dana.marekova@iem.cas.cz (D.M.); 2 Institute of Macromolecular Chemistry, Czech Academy of Sciences, Heyrovského nám. 2, 16200 Prague, Czech Republic; vasylyshyn@imc.cas.cz (T.V.); patsula@imc.cas.cz (V.P.); oleksa@imc.cas.cz (V.O.); shapoval@imc.cas.cz (O.S.); horak@imc.cas.cz (D.H.); 3 Center for Advanced Preclinical Imaging, First Faculty of Medicine, Charles University, Salmovská 3, 12000 Prague, Czech Republic; petr.matous@lf1.cuni.cz (P.M.); vit.herynek@lf1.cuni.cz (V.H.); 4 Department of Analytical Chemistry, University of Chemistry and Technology, Technická 5, 16000 Prague, Czech Republic; magda.vosmanska@vscht.cz; 5 Imaging Methods Core Facility, BIOCEV, Faculty of Science, Charles University, Průmyslová 595, 25250 Vestec-Jesenice u Prahy, Czech Republic; liebl@natur.cuni.cz (D.L.); ales.benda@natur.cuni.cz (A.B.)

**Keywords:** upconverting nanoparticles, toxicity, biological applications

## Abstract

In this study, spherical or hexagonal NaYF_4_:Yb,Er nanoparticles (UCNPs) with sizes of 25 nm (S-UCNPs) and 120 nm (L-UCNPs) were synthesized by high-temperature coprecipitation and subsequently modified with three kinds of polymers. These included poly(ethylene glycol) (PEG) and poly(N,N-dimethylacrylamide-co-2-aminoethylacrylamide) [P(DMA-AEA)] terminated with an alendronate anchoring group, and poly(methyl vinyl ether-co-maleic acid) (PMVEMA). The internalization of nanoparticles by rat mesenchymal stem cells (rMSCs) and C6 cancer cells (rat glial tumor cell line) was visualized by electron microscopy and the cytotoxicity of the UCNPs and their leaches was measured by the real-time proliferation assay. The comet assay was used to determine the oxidative damage of the UCNPs. An in vivo study on mice determined the elimination route and potential accumulation of UCNPs in the body. The results showed that the L- and S-UCNPs were internalized into cells in the lumen of endosomes. The proliferation assay revealed that the L-UCNPs were less toxic than S-UCNPs. The viability of rMSCs incubated with particles decreased in the order S-UCNP@Ale-(PDMA-AEA) > S-UCNP@Ale-PEG > S-UCNPs > S-UCNP@PMVEMA. Similar results were obtained in C6 cells. The oxidative damage measured by the comet assay showed that neat L-UCNPs caused more oxidative damage to rMSCs than all coated UCNPs while no difference was observed in C6 cells. An in vivo study indicated that L-UCNPs were eliminated from the body via the hepatobiliary route; L-UCNP@Ale-PEG particles were almost eliminated from the liver 96 h after intravenous application. Pilot fluorescence imaging confirmed the limited in vivo detection capabilities of the nanoparticles.

## 1. Introduction

Since their first development in the early 2000s, lanthanide-doped upconversion nanoparticles (UCNPs) have attracted extensive attention in various cutting-edge bioapplications such as deep tissue bioimaging, biosensing, and nanomedicine [1,2]. The ability of UCNPs to convert incident light in the near-infrared (NIR) region into high-energy ultraviolet or visible emission via an anti-Stokes process has been exploited [3,4]. This allows not only relatively deep light penetration and low photodamage effects, but also reduced autofluorescence, light scattering, and phototoxicity, enabling simultaneous applications of luminescent nanomaterials in areas such as precise theranostics, in vivo optogenetics, and environmental hazard control [5,6,7]. Important prerequisites for the use of UCNPs in biomedical applications are hydrophilicity and dispersibility in biological buffers, together with chemical and colloidal stability and high signal reproducibility [8]. These properties are exhibited by homogeneous phase-pure particles prepared under specific conditions. Other characteristics of UCNPs influencing their use are biocompatibility and non-toxicity, which are affected by particle size, shape, chemical composition, and surface structure [9,10]. Dissolution of UCNPs in aqueous media can induce cytotoxicity in biological systems, especially under conditions of high dilution in aqueous media [11,12,13,14]. A tendency of Ln fluorides to dissolve in phosphate buffers due to the release of Ln^3+^ and F-ions, which can lead to a gradual loss of luminescence intensity, uneven particle brightness, and even localized cytotoxicity, has been recently confirmed [15]. In addition, the release of Y^3+^ ions can affect geno- and/or cytotoxicity and possible neuronal damage [16], which is a barrier for medical applications [13]. The toxicity assessment of UCNPs is therefore a relevant issue since many researchers are using them for various applications, such as bioimaging or drug delivery. There are several methods for producing rare earth-doped UCNPs, e.g., high-temperature coprecipitation [17], thermal decomposition [18], and hydro(solvo)thermal [19], microwave [20], or microemulsion synthesis [21]. Among these procedures, the most popular preparation of monodisperse crystalline UCNPs of various sizes and compositions is the aforementioned “user-friendly” coprecipitation using oleic acid as a stabilizer [22]. By choosing the reaction parameters, either small spherical or large hexagonal particles can be obtained. Small particles are generally well colloidally stable, have less tendency to become trapped in the reticuloendothelial system and thus exhibit long blood circulation time, and are thus suitable for drug delivery systems or photodynamic therapy of tumors. In contrast, large hexagonal particles often provide higher luminescence intensity than small particles; thus, these large particles are mainly used in biosensing (detection of biomarkers of various diseases from blood).

Recently, some studies have appeared dealing with the protection of UCNPs by introducing a surface layer that provides both their colloidal stability and insolubility in aqueous media, as well as suppresses luminescence quenching and minimizes particle toxicity [14,23]. The advantage of UCNP surface modification is also the possibility of introducing functional groups allowing the attachment of a large number of biomolecules such as peptides, proteins, antibodies, DNAs, drugs, photosensitizers, etc., which facilitate specific targeting and treatment. These functional groups consist of carboxyl, amino, thiol, maleimide, aldehyde, phosphate, bisphosphonate, sulfonate, and even o-nitrobenzyl groups [24]. General surface engineering strategies of UCNPs include ligand oxidation, replacement, or removal of hydrophobic stabilizers, as well as silanization, layer-by-layer assembly, coating with amphiphilic polymers, etc. [25]. This has allowed the modification of UCNPs with poly(ethylene glycol) (PEG) and its derivatives [26,27], amphiphilic chitosan [28], poly(acrylic acid) [29], polyethyleneimine [30], polyvinylpyrrolidone [31], poly(D,L-lactic-co-glycolic acid) [32], poly(isobutylene-alt-maleic anhydride) [33], etc. The surface design of UCNPs can also be optimized by encapsulating them in polymers via microemulsion polymerization of monomers such as ethylene glycol methyl ether acrylate, 2-hydroxyethyl and glycidyl methacrylate, acrylic acid, or by “grafting-from” and “grafting-on” methods [34,35]. The quality of the surface composition is a key factor affecting the circulation time of UCNPs in the bloodstream and/or passing through cell membranes, which is crucial for their bioapplications. The recommended size for optimal UCNP penetration is less than 100 nm. However, this size may also pose a risk of toxicity due to their potential to penetrate cellular structures and organs via the circulatory system. In addition, UCNPs can generate reactive oxygen species (ROS) that can induce DNA damage, which can lead to damage not only affecting cell growth through protein oxidation, but also impacting mitochondrial respiration [36]. Nanoparticles administered intravenously circulate through the bloodstream, especially to the liver and spleen, kidneys, heart, lungs, bone marrow, and brain. Retention in the blood and organs strongly depends on the surface properties of the nanoparticle material. Coatings that promote interactions of NPs with cell membranes promote internalization by different cell types, while the use of biologically inert coatings, i.e., PEG, leads to prolonged circulation of these NPs in the bloodstream. Moreover, the rate and mechanism of uptake and clearance is cell/tissue-dependent and varies between NPs of different hydrodynamic size, composition, shape, charge, and surface functional groups [37,38].

In this work, we prepared NaYF_4_:Yb,Er-based UCNPs and investigated the dependence of their morphology, size, and type of coating on their cytotoxicity as determined on rMSCs and C6 cells using the xCELLigence real-time proliferation assay. We also investigated their internalization in cells and DNA oxidative damage caused by coated and uncoated, small, and large UCNPs. As a proof of concept, UCNPs were also tested in experimental animals and imaged in vivo.

## 2. Results and Discussion

**Figure 1:**
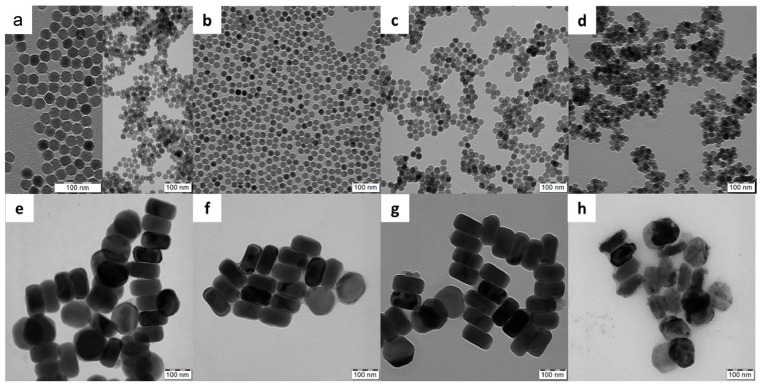
TEM micrographs of (**a**–**d**) small spherical-like and (**e**–**h**) large hexagonal UCNPs; (**a**,**e**) non-coated and coated UCNPs with (**b**,**f**) Ale-PEG, (**c**,**g**) Ale-(PDMA-AEA), and (**d**,**h**) PMVEMA. The higher magnification of the spherical-like shape is shown in (**a**).

This work is a continuation of our previous papers, in which we investigated the design and properties of small and large surface-engineered UCNPs with emphasis on their colloidal and chemical stability [39,40,41]. In this report, we compared the two types of UCNPs, small spherical (S-UCNPs) and large hexagonal (L-UCNPs), prepared by high-temperature coprecipitation of the respective lanthanide chlorides depending on the reaction conditions (Figure 1), in terms of morphology, cytotoxicity, and detection of DNA damage by the comet assay. While the spherical particles were 25 nm in diameter, their hexagonal counterparts were ~120 nm in size; both types had a relatively narrow particle size distribution (*Đ* = 1.01), which is essential for biomedical applications as it allows control of particle properties and reproducibility of results [39]. Moreover, three non-toxic biocompatible polymer coatings, namely Ale-PEG, Ale-P(DMA-AEA), and PMVEMA, were selected to ensure the colloidal stability of the particles in media [40]. The properties of these coatings differed; while nonionic Ale-PEG is known for its antifouling properties [42], the positively charged Ale-P(DMA-AEA) coating of UCNPs promoted their engulfment by cells [43]. The latter coating also has the advantage of the presence of reactive amino groups available for prospective attachment of different biomolecules. Both Ale-PEG and Ale-(PDMA-AEA) were terminated with a bisphosphonate group that forms highly stable metal-bisphosphonate complexes firmly anchoring the polymers to the particle surface. In contrast, negatively charged PMVEMA has multiple carboxyl groups that can coordinate with lanthanide surface ions and/or react with various compounds.

Because all polymers did not exhibit phase contrast in the TEM images, the TEM micrographs of the polymer-coated UCNPs did not differ from those of the starting uncoated particles (Figure 1). The hydrodynamic diameter (*D*_h_) of S-UCNPs and S-UCNP@Ale-PEG, S-UCNP@Ale(PDMA-AEA), or S-UCNP@PMVEMA nanoparticles was 101, 68, 102, and 112 nm, respectively. Their ζ-potential, which depends on the surface chemistry of the particle, was 30, 8, 27, and −32 mV for neat, Ale-PEG-, Ale(PDMA-AEA)-, and PMVEMA-modified particles, respectively (Table 1). All studied L-UCNPs had comparable ζ-potential as that of S-UCNPs, but due to their larger size (according to TEM) they had a larger *D*_h_. The resulting *D*_h_ values of L-UCNP, L-UCNP@Ale-PEG, L-UCNP@Ale(PDMA-AEA), and L-UCNP@PMVEMA nanoparticles were 174, 158, 160, and 234 nm, respectively, and the corresponding ζ-potential was 28, 4, 22, and −46 mV, respectively (Table 1). Monitoring the fluoride ions dissolved in media in which the particles were aged showed that their degradation depended on many parameters, including size, coating type, temperature, and the medium used [40,41]. When released, Ln^3+^ and F^−^ ions engage with cellular phosphates found in membranes, ATP, and nucleic acids, resulting in cell damage. The toxicity escalates with higher dissolution rates. Therefore, it is necessary to encapsulate UCNPs with biocompatible polymers to mitigate these effects [39,40]. All types of selected coatings were shown to decrease particle degradation in different aqueous media, with the exception, of PMVEMA, which slightly increased particles’ dissolution in water. It was also shown that dissolution of UCNPs was low in water and DMEM, moderate in artificial lysosomal fluid, and pronounced in PBS. Increasing temperature directly increased dissolution of particles regardless of their size, coating, and aging medium. Moreover, independently of the type of coating, te L-UCNPs dissolved less than the S-UCNPs due to a smaller surface-to-volume ratio. This observation was also confirmed by the ICP-MS, where the amount of Y and Yb was measured in leaches. The results showed that S-UCNPs were more soluble in culture media (Y and Yb within the range of 2–8 µg/mL) compared to the L-UCNPs, where both elements were present at less than 2 µg/mL. Therefore, parameters like particle size, ζ-potential, and storage medium can influence particle cytotoxicity due to the interaction of the particles or their degradation products with cells. Our results are in an agreement with other studies in which the stability of NaYF_4_:Yb^3+^,Er^3+^ UCNPs stabilized with phosphonate coatings alendronate and ethylenediamine tetra(methylene phosphonic acid) in PBS or culture medium at room temperature and 37 °C was also investigated [6,27,44,45].

### 2.1. Internalization of UCNPs in Cell Cytoplasm

**Figure 2:**
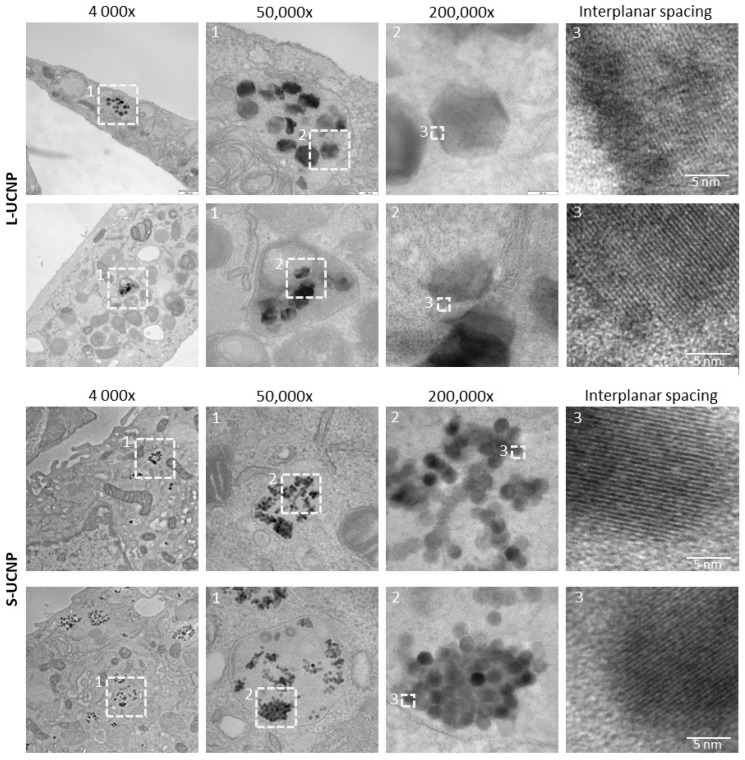
Ultrastructural TEM analysis of nanoparticle intracellular localization. Two representative pictures (lower vs. upper panel) are shown for L-UCNPs and S-UCNPs internalized in rMSCs.

Both types of nanoparticles (L-UCNPs and S-UCNPs) were internalized into membrane-delimited endosomal compartments with noticeable aggregation of nanoparticles into clusters within the lumen of endosomes in rMSCs (Figure 2). Lower magnifications (4000× and 50,000×) demonstrated the position of particle-containing endosomes within the cell, and HRTEM images (200,000×) demonstrated the crystalline structure of nanoparticles with discernible interplanar spacing (Figure 2, enlarged on the right). Various densities of L-UCNP nanoparticles (an average diameter of 120 nm) and S-UCNP nanoparticles (an average diameter of 25 nm) were found within the section of 60 nm. The observation of UCNPs being internalized into membrane-delimited endosomal compartments is consistent with known cellular uptake mechanisms for nanoparticles. Cells often internalize nanoparticles through endocytic pathways, including clathrin-mediated endocytosis, caveolae-mediated endocytosis, and macropinocytosis. These pathways involve the formation of vesicles at the cell membrane, which encapsulate extracellular materials, including nanoparticles, and transport them into the cell’s interior [46,47].

Furthermore, the aggregation of nanoparticles into clusters within the lumen of endosomes is a common phenomenon observed in nanoparticle–cell interactions. This aggregation can occur due to various factors, such as electrostatic interactions between nanoparticles, steric hindrance, and protein corona formation. The presence of nanoparticle clusters within endosomes may influence their intracellular trafficking and fate, potentially affecting their subsequent release or degradation within the cell [48].

The characterization of nanoparticle internalization using TEM allows for the detailed visualization of nanoparticles within cellular compartments. High-resolution TEM images provide insights into the crystalline structure of nanoparticles, revealing features such as lattice spacing and crystal orientation. This information is valuable for understanding the stability and integrity of nanoparticles within the cellular environment.

### 2.2. Cytotoxicity by xCELLigence Assay

**Figure 3:**
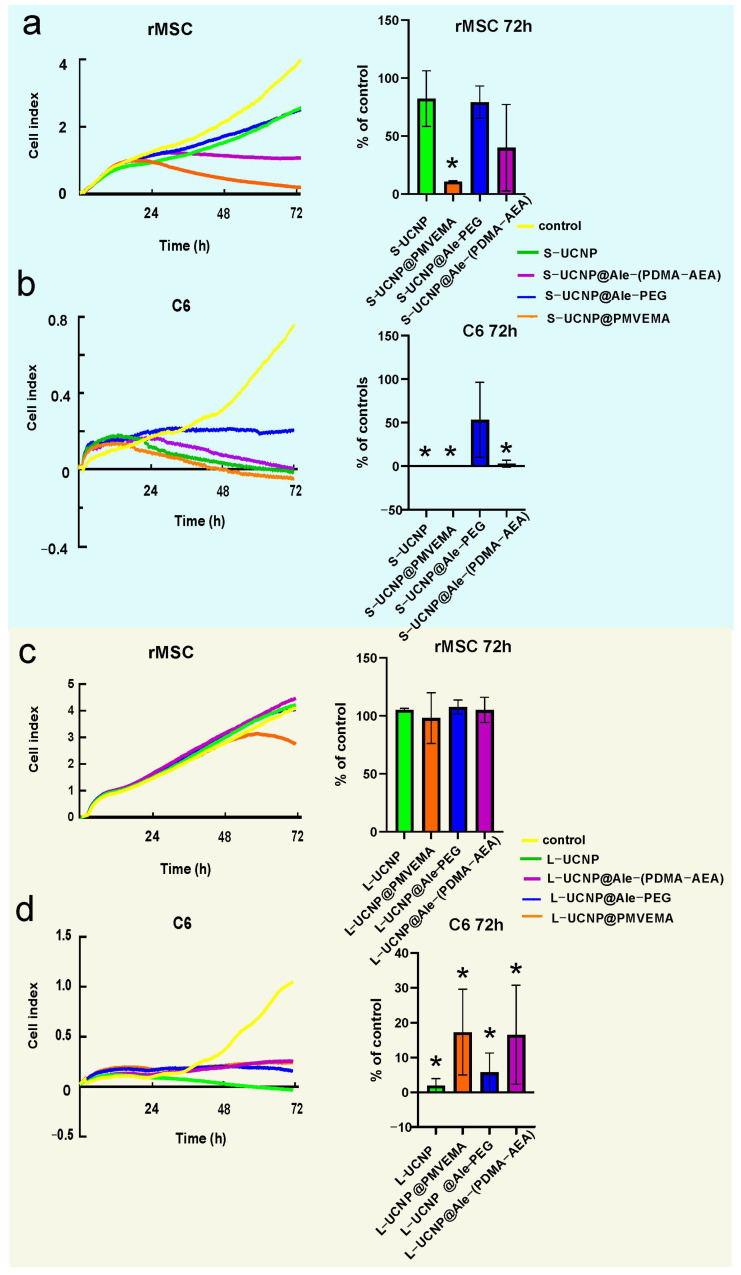
Proliferation curves of rMSCs and C6 cells incubated with (**a**,**b**) small and (**c**,**d**) large UCNPs for 7 h. * *p* ≤ 0.05.

**Figure 4:**
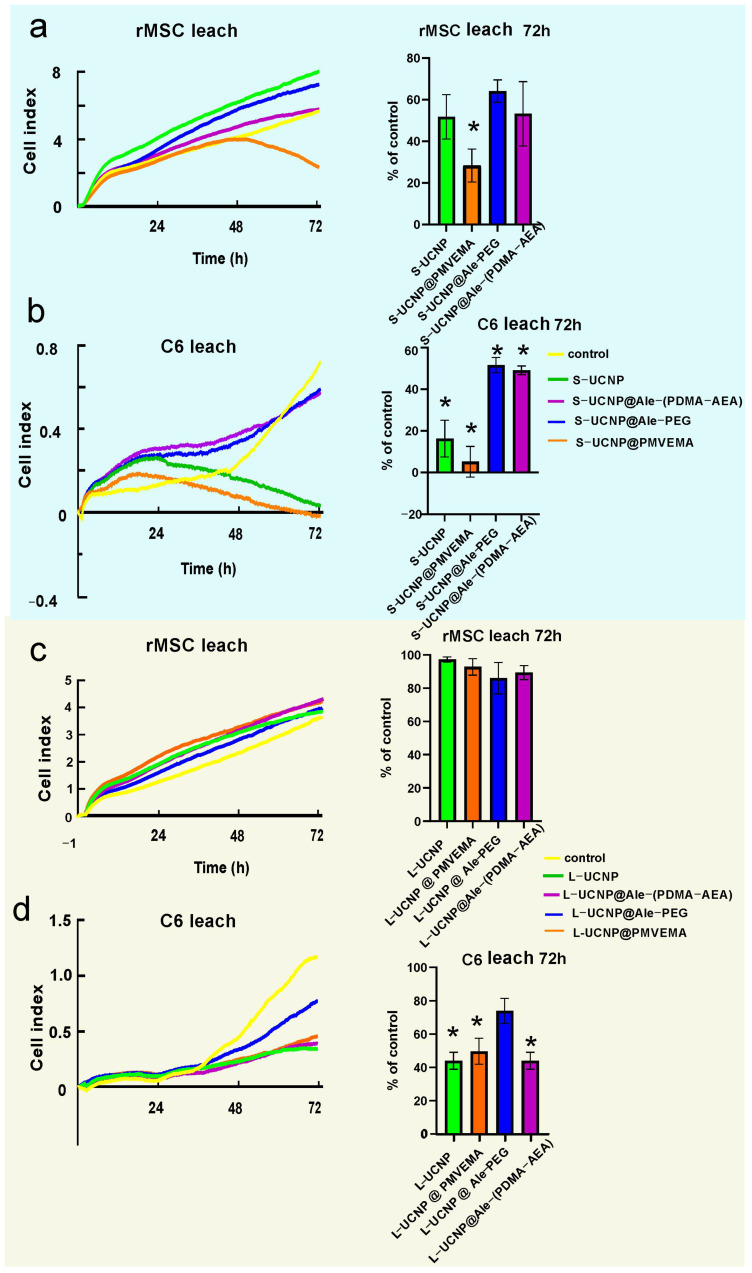
Proliferation curves of rMSCs and C6 cells incubated with leaches from (**a**,**b**) S-UCNPs and (**c**,**d**) L-UCNPs for 72 h. * *p* ≤ 0.05.

To monitor cell growth dynamics, the viability of C6 and rMSCs incubated with Ale-PEG-, Ale-(PDMA-AEA), and PMVEMA-coated L- and S-UCNPs for 3 days was examined using a real-time proliferation assay. Compared to traditional endpoint assays such as the MTT assay or LDH release assay, the xCELLigence system offers the advantage of real-time monitoring of cytotoxicity, enabling the dynamic assessment of cellular responses over time without the need for exogenous labels or dyes, thus providing richer and more continuous data on cellular behavior. Additionally, its high throughput and automation capabilities make it particularly well-suited for large-scale studies and high-content screening applications, enhancing experimental efficiency and reproducibility. The test is performed in wells coated with gold electrodes, where cells’ growth is expressed as a cell index correlating with an increase in electrical impedance. Cell proliferation curves were determined after incubation with both particle types and their leaches (Figure 3 and Figure 4). Particles were used at a concentration of 20 µg/mL, at which the viability of C6 and rMSCs in previous experiments using the Alamar blue or MTT assay reached >80% [40,41]. The growth of healthy non-tumor rMSCs incubated with any type of UCNPs for 1 day was similar, to the control. After 2 days of incubation of rMSCs with S-UCNP@PMVEMA, there was a dramatic decrease in cell proliferation, which continued for the next 24 h (*p* = 0.031). In the presence of S-UCNP@Ale-(PDMA-AEA) particles, cell growth decreased slightly, then remained unchanged over 72 h (Figure 3a). Cell viability in the presence of S-UCNP@Ale-PEG and neat S-UCNPs remained ~80%. We speculate that uncoated S-UCNPs partially aggregated and thus were not uniformly dispersed in the well. Therefore, the cytotoxicity was lower than that of the S-UCNP@Ale-(PDMA-AEA) particles. In contrast, all types of L-UCNPs had no effect on cell growth, except L-UCNP@PMVEMA, where rMSC proliferation decreased after 48 h of incubation (Figure 3c). In general, S-UCNPs impaired cancer C6 cell viability more significantly than rMSCs. While control cells proliferated rapidly for all 3 days, C6 cells incubated with S-UCNPs for 1 day stopped growing. The viability of C6 cells in the presence of S-UCNP@Ale-PEG particles remained constant but decreased after incubation with neat S-UCNPs for 72 h (*p* = 0.0142) or their analogues coated with Ale-(PDMA-AEA) (*p* = 0.0162) or PMVEMA (*p* = 0.0142; Figure 3b). Similar results were achieved with L-UCNPs, especially neat ones, which stopped the proliferation of C6 cells that detached from the bottom of the well (Figure 3d). Prolonged exposure to all polymer-coated L-UCNPs (72 h) at a concentration of 20 µg/mL reduced cell growth (*p* < 0.0001) as did neat L-UCNPs (*p* < 0.0001). Our results are in an agreement with MTS assay studies that reported a 67% decrease in viability of bone marrow-derived stem cells after 48 h of exposure to UCNPs [8,49] while shorter incubation times did not affect cell viability. Also, UCNPs coated with polyethylenimine (25 µg/mL) exposed to MSCs for 2 days slightly decreased cell viability to 85% [50]. Different coatings of UCNPs were also tested on HaCaT keratinocytes by the WST-8 assay and, similarly, prolonged exposure to UCNPs for 48 h resulted in a viability drop when compared to 24 h [11]. In contrast to nanoparticles, rMSCs incubated with particle leaches from neat S-UCNPs, S-UCNP@PMVEMA, S-UCNP@Ale-(PDMA-AEA), and S-UCNP@Ale-PEG proliferated even faster than in the control group (Figure 4a), indicating stimulation of cell growth. In the case of C6 cells, their growth was faster after incubation with leaches for 24 h; in later time periods (72 h), leaches from neat S-UCNPs (*p* = 0.0015), S-UCNP-Ale-PEG (*p* = 0.0119), S-UCNPs@Ale-(PDMA-AEA) (*p* = 0.0098), and S-UCNP@PMVEMA (*p* = 0.0009) were toxic (Figure 4b). On the other hand, the leaches from L-UCNPs did not affect rMSC proliferation (Figure 4c); however, C6 growth was slowed down after the incubation with L-UCNP leaches for 72 h (*p* = 0.0086), L-UCNP@PMVEMA leaches (*p* = 0.0129), and L-UCNP@Ale-(PDMA-AEA) leaches (*p* = 0.0086), except for L-UCNP@Ale-PEG leaches, where the cell proliferation decreased only marginally (Figure 4d).

Thus, it can be concluded that the real-time proliferation assay in cells incubated with UCNPs gave similar results to the MTT or Alamar blue assay performed in previous experiments [40,41], while the leaches, especially from L-UCNPs, were less harmful. In general, L-UCNPs were less toxic than S-UCNPs due to the lower surface-to-volume ratio. L-UCNP nanoparticles almost did not affect the viability of rMSCs, while the viability of C6 cells significantly decreased to 30% or less. The viability of rMSCs decreased in the order S-UCNP@Ale-(PDMA-AEA) > S-UCNP@Ale-PEG > S-UCNPs > S-UCNP@PMVEMA. The trend was also similar for C6 cells, which readily internalized particles depending on the incubation time. In contrast, rMSCs engulfed particles much less after three days of incubation than after one day [40]. The best biocompatibility was achieved with Ale-(PDMA-AEA) and Ale-PEG coatings, which can be attributed to their hydrophilicity. There are alternative approaches to assess NP cytotoxicity: for example, Das et al. [51] conducted a study on the toxic effects of three types of functionalized UCNPs: oleate ligand-UCNPs, PEG-UCNPs, and bilayered PEG-oleate-UCNPs. They used calcein and a propidium iodide viability assay and concluded that bilayer UCNPs exhibit significant toxicity due to functionalization. In another study, Malvindi et al. [52] evaluated the cytotoxicity of silica-coated iron oxide NPs using the WST-8 [2-(2-methoxy-4-nitrophenyl)-3-(4-nitrophenyl)-5-(2,4-disulfophenyl)-2H-tetrazolium, monosodium salt] method and the lactate dehydrogenase release assay (LDH assay) to analyze cell viability and cell membrane integrity. The NPs showed good internalization in HeLa cells with no observed toxicity. Meindl et al. [53], on the other hand, assessed the cytotoxicity of UCNPs by measuring intracellular calcium, providing an example of an alternative approach to assess toxicity. 

### 2.3. Genotoxicity by Comet Assay

**Figure 5:**
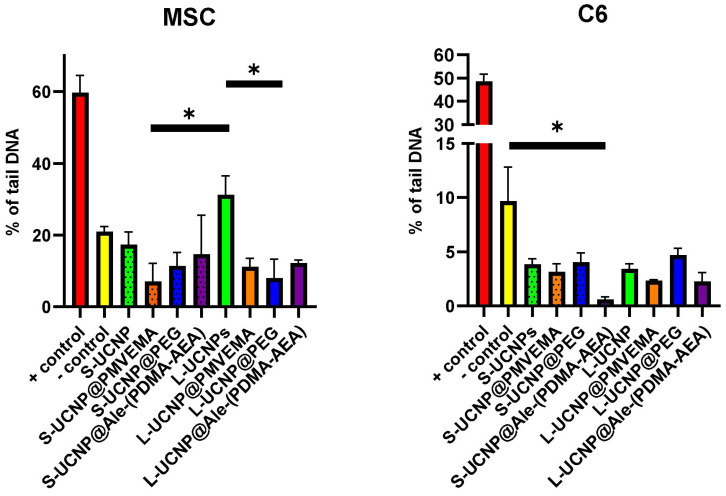
The oxidative damage of UCNPs expressed as a percentage of tail DNA from rMSCs and the C6 cell line. The statistical difference was found between L-UCNPs vs. L-UCPN@Ale-PEG and S-UCNP@PMVEMA in rMSCs; in C6 cells, no difference was found when comparing different nanoparticle treatments. The difference was found between S-UCNP@Ale-PDMA and the negative control. * *p* ≤ 0.05.

In rMSCs, all nanoparticle treatments were statistically significant to the positive control and non-significant to the negative control. In addition, when comparing oxidative damage of large vs. small UCNPs and coated vs. uncoated UCNPs on rMSCs, a statistically significant difference was found between L-UCNPs vs. L-UCNP-Ale-PEG (*p* = 0.021) and S-UCNP@Ale-PMVEMA (*p* = 0.0158). In C6 cells, no difference was found when comparing different nanoparticle treatments. The difference was found between S-UCNP@Ale-(PDMA-AEA) and the negative control (*p* = 0.0258; Figure 5). In both rMSCs and C6 cells, the negative and positive controls were statistically significant (*p* < 0.001). In general, oxidative damage was ~20% for rMSCs regardless of nanoparticle treatment, while it was only ~5% for C6 cells. Thus, C6 cells were less sensitive to oxidative damage than rMSCs under controlled conditions, which can be explained by the reduced sensitivity of the cell lines to higher oxygen levels under normal in vitro incubation conditions than in animal tissues. Similar results have been reported on the UCNPs based on Y_2_O_3_/Yb^3+^,Er^3+^ where no DNA damage was found on the cancer cells in the comet assay [54]. On the contrary, a lower viability of C6 cells in the presence of UCNPs may be explained by the smaller cell volume relative to the number and size of internalized nanoparticles. The differences between rMSCs and C6 cells are also due to the cell origin, were there are big differences in cell proliferation and growth, differentiation potential, function, role, and genetic and molecular characteristics. Changes observed in the comet assay following exposure to UCNPs may result from direct interactions between UCNPs and DNA, leading to physical damage or cross-linking. Additionally, UCNPs may induce the generation of reactive oxygen species (ROS), causing oxidative damage to DNA, which is detectable as increased DNA fragmentation. The size, surface properties, and concentration of UCNPs, along with cell type specificity, contribute to the variability in comet assay responses. These results confirm that functionalized UCNPs can be used without any genotoxic effects for bioimaging.

### 2.4. In Vivo Study

**Figure 6:**
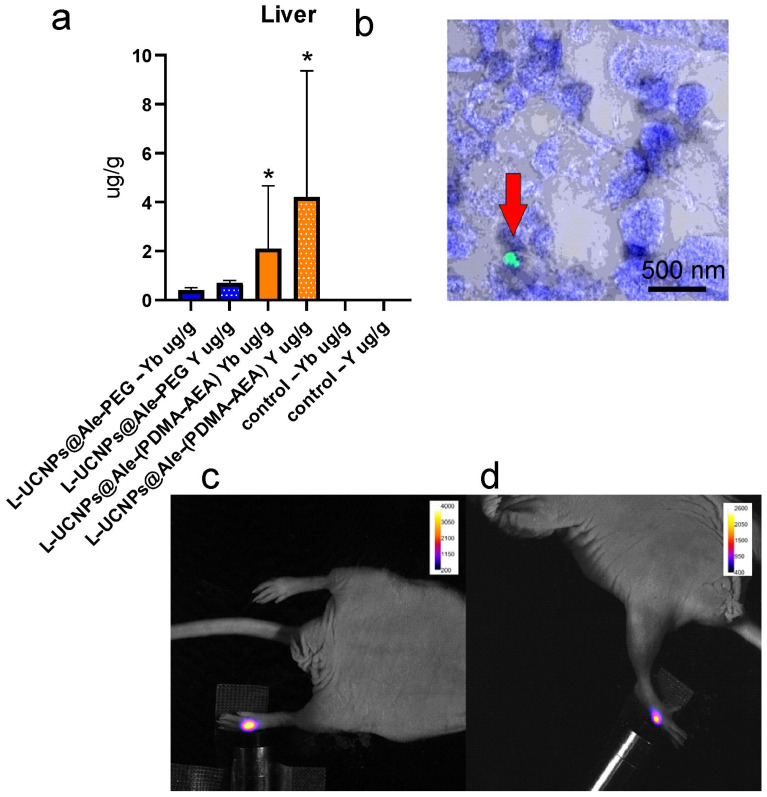
(**a**,**b**) The intravenous application of the L-UCNP@Ale-PEG and L-UCNP@Ale-(PDMA-AEA) particles and saline. (**a**) L-UCNP@Ale-PEG particles were almost completely eliminated from the liver 96 h after application, while L-UCNP@Ale-(PDMA-AEA) particles remained there in a significantly higher amount. (**b**) The L-UCNP@Ale-(PDMA-AEA) particles were detected in the liver using two-photon microscopy (the arrow shows L-UCNP@Ale-(PDMA-AEA) particles in the liver tissue (**b**)). A fluorescence signal at 535 nm of subcutaneously administered (**c**) L-UCNP@Ale-PEG and (**d**) L-UCNP@Ale-(PDMA-AEA) particles was detected in vivo in an experimental animal after excitation at 980 nm. * *p* ≤ 0.05.

Nanoparticles were investigated in vivo after subcutaneous application in NuNu mice (males, 6–7 weeks old). Upconversion enabled the detection of a signal at 535 nm after excitation in the near-infrared area (980 nm; Figure 6). After systemic (retroorbital) application, in vivo imaging failed to detect the particles. While near-infrared excitation light easily penetrates the tissue and may excite, weaker fluorescent light at 535 nm is absorbed. Therefore, the possibility to obtain the fluorescence signal at this wavelength from organs located deep in the body (e.g., the liver) is very limited. The nanoparticles were detected postmortem in the excised organs. Fluorescence microscopy proved the presence of L-UCNP@Ale-(PDMA-AEA) in the liver only (Figure 6). L-UCNP@Ale-PEG particles were not detected in any organ. These results were supported by ICP-MS, which confirmed the presence of Yb and Y atoms from the nanoparticles only in the liver. The amount of Yb and Y detected from Ale-PEG-coated UCNPs was significantly lower than that from Ale-(PDMA-AEA)-coated nanoparticles (*p* < 0.0001), where a statistically significant difference was observed compared to the control group. A statistically significant difference between UCNP@Ale-PEG and the control group was not observed; the particles were almost completely eliminated from the body after more than 96 h (Figure 6a). No Yb and Y atoms were detected by ICP-MS in the kidneys. These experiments confirmed the fast elimination of the coated UCNPs from the organism and suggested the hepatobiliary excretion route. The hepatobiliary excretion route involves several processes: phagocytosis by Kupffer cells, diffusion through liver sinusoidal endothelial cell fenestrae to the space of Disse, metabolization of toxins by hepatocytes, and drainage by bile ducts. Excretion substantially depends on the size of nanoparticles [55]. Smaller particles are eliminated faster, as they have easier access to the space of Disse. Larger particles are phagocyted by Kupffer cells and degraded or metabolized before their elimination, however, their elimination is much slower. Particle size may be responsible for the differences in elimination of Ale-PEG-coated and Ale-PDMA-coated particles; Ale-PEG particles have a smaller hydrodynamic diameter (see Table 1), so therefore, their excretion is faster. The UCNPs coated with PEG were eliminated from the body faster than PDMA-AEA-coated UCNPs. As was discussed in the review by Zhang [56], the PEG-coated formulations tend to display enhanced solubility, prolonged circulatory time, and reduced immunogenicity/antigenicity, which was also confirmed in our study. Contrary to this, Zhou et al. [57] observed a tendency for NaYF_4_:Yb,Er@SiO_2_ to accumulate in the liver after entering the bloodstream. A large amount of NaYF_4_:Yb,Er@SiO_2_ was observed in the liver tissue on days 1 and 7 after the intravenous administration of these nanoparticles to mice at a dose of 20 mg/kg, suggesting internalization into hepatocytes. However, the dose was approximately 10 times higher than in our experiment. Even after the application of this high dose, the histology did not reveal any pathological changes in the liver tissue.

It has been shown that upconverting nanoparticle detection in vivo is challenging. The red excitation light penetrates deep into the mouse tissue, but the green emission light is absorbed by the tissue itself (namely, blood absorbs light at this wavelength). In subcutaneous application, the green light emitted by the nanoparticles is easy to detect due to the locally high nanoparticle concentration and shallow depth. However, a pilot experiment confirmed that light intensity at this wavelength is not sufficient for the imaging of nanoparticle distribution after systemic application. Moreover, the laser diode is suitable for local in situ excitation of the nanoparticles, but it cannot be used for irradiation of the whole animal and reliable monitoring of the distribution within the whole body.

In vivo imaging of UCNPs also has other limitations that affect the experimental outcome. The laser diode we used for the experiment had no tuneable power and had a power of 100 mW at an inner diameter of 3.55 mm. In future experiments, it would be preferable to use a laser with a tuneable power to maximize the quantum yield of the upconversion and to ensure a safe dose of radiation at the same time.

The effect of coating on reducing UCNP cytotoxicity has already been reviewed [41]. This publication showed that uncoated nanoparticles had a substantial effect on cell viability. Nanoparticle coating suppressed the release of lanthanide Yb^3+^ and Er^3+^ from nanoparticle cores, which are potentially toxic. However, the coating may also have a negative effect on the quantum yield of upconversion. Thus, the choice of a suitable coating is crucial and both phenomena (biocompatibility and upconversion factors) should be taken into account.

While fluorescence particle detection in vivo is very difficult, it does not compromise future use of UCNPs in photodynamic therapy in combination with a suitable photosensitizer. If the particles are carefully navigated, they can be excited by NIR light deep in the tissue. Generated fluorescence light can irradiate an immediate vicinity only, so it may excite a suitable photosensitizer locally with no possible harm to other tissues or organs.

## 3. Materials and Methods

Small (S) spherical and large (L) hexagonal UCNPs were prepared by high-temperature coprecipitation of lanthanide chlorides and their surface was modified with poly(ethylene glycol) (PEG) and poly(N,N-dimethylacrylamide-co-2-aminoethylacrylamide) [P(DMA-AEA)], both terminated with an alendronate (Ale) anchoring group, and poly(methyl vinyl ether-co-maleic acid) (PMVEMA), according to our previous work [40,41]. For cell culturing phosphate-buffered saline (PBS), Dulbecco’s modified Eagle medium (DMEM), supplemented with fetal bovine serum (FBS) (both from Merck; Darmstadt, Germany), a combination of primocin and penicillin–streptomycin (Gibco; Life Technologies, Grand Island, NY, USA) and trypsin (Sigma-Aldrich, St. Louis, MO, USA), was used. For the preparation of samples for electron microscopy, we utilized PHEM buffer (pH 7.4) OsO_4_ solution (Thermo Fisher, Waltham, MA, USA), K_3_(Fe(CN_6_)) (VWR International, Radnor, PA, USA), low-melting agarose (LMP) (Sigma-Aldrich, St. Louis, MO, USA), uranyl acetate (Sigma-Aldrich, St. Louis, MO, USA), and Epon HARD complete resin (Electron Microscopy Sciences; Hatfield, PA, USA); for fluorescent microscopy, 4′,6-diamidino-2-phenylindole dihydrochloride (DAPI; Invitrogen; Carlsbad, NM, USA) was obtained.

All other chemicals were purchased from Sigma-Aldrich, or LachNer (Neratovice, Czech Republic).

Distilled and demineralized water (conductivity ˂ 0.1 µS/cm; Millipore; Bedford, MA, USA) was used to prepare all solutions.

### 3.1. Preparation of Leachates

For toxicity analysis of the leachates, these were prepared from dispersions of particles in the culture medium. The nanoparticles were mixed with the medium to a concentration of 20 µL/mL, the mixture was placed in an incubator at 37 °C for three days, the dispersion was centrifuged (160 RCF) for 5 min, and the supernatant was analyzed using a real-time proliferation assay.

### 3.2. C6 Cell Line

To initiate the cell culture, C6 cells (C6 cell line from rat, Cat. No 92090409, Sigma-Aldrich, St. Louis, MO, USA) were thawed and rinsed in cold PBS. After washing, they were seeded in DMEM and supplemented with FBS and a combination of primocin and penicillin–streptomycin.

### 3.3. Mesenchymal Stem Cells

Rat mesenchymal stem cells (rMSCs) were derived by aspirating bone marrow from the rat’s bone. The harvested bone marrow was subsequently washed twice with PBS. The rinsed bone marrow was then cultured in cell culture flasks containing DMEM. The culture medium was enriched with FBS, along with the addition of primocin and penicillin–streptomycin. The culture flasks were placed in an incubator at 37 °C and 5% CO_2_ atmosphere and the culture medium was changed twice a week. Upon reaching approximately 70% confluence, the cells were passaged using trypsin at 37 °C for 4 min. Trypsin treatment was terminated by FBS. The cells were subsequently washed with PBS and either utilized for the experiment or reseeded for further culture. Both cell cultures were maintained at 37 °C in a 5% CO_2_ atmosphere. The culture medium was refreshed twice a week to maintain cell viability and growth.

### 3.4. UCNPS Treatment

For all experiments, 20 μg/mL concentrations of all types of nanoparticles were used. The UCNPs were added to the culture media after reaching 70% cell confluence. The cells were treated for the whole incubation time—72 h in the case of the xCELLigence assay and for 24 h in the case of the TEM and comet assay.

### 3.5. Electron Microscopy

The internalization of the nanoparticles into the C6 cells and MSCs was investigated by transmission electron microscopy (TEM). The cells were cultured up to the 70% confluence, and L-UCNPs and S-UCNPs were added for 24 h to the culture media as described before. The cells were then collected, initially centrifuged at 300× *g* for 5 min at room temperature (RT). They were then fixed with a solution containing 2.5% glutaraldehyde, 2% formaldehyde, and 0.1 M PHEM buffer (pH 7.4) for 10 min at RT and incubated on ice for 1 h. After fixation, the samples underwent three 5 min ice-cold washes with 0.1 M PHEM buffer (pH 7.4).

Following the washing, the samples were contrasted in 1% OsO_4_ solution and 1.5% K3(Fe(CN6)) in Milli-Q water on ice for 1 h. The samples were washed three times for 5 min each with PHEM buffer (pH 7.4) while kept on ice. Subsequently, the samples were centrifuged at 400 g for 2–5 min at 38 °C, embedded in 2% LMP at 38 °C, and polymerized for 20 min on ice. Afterward, cubes were cut from the samples, quickly washed with cold Milli-Q water, and stained in a 1% uranyl acetate aqueous solution at RT for 30 min. Dehydration involved washing with a series of cold ethanol solutions (30–50–70–80%) for 5 min each on ice, followed by RT-warmed 90–100% ethanol for 5 min at RT. The samples were then transferred into anhydrous acetone at RT, infiltrated and embedded in epoxy resin kit EMBED 812 (EMS#14120), and polymerized at 40 °C for 1 h and at 60 °C for 72 h in an oven. Ultrathin sections were cut on a LEICA UC7 ultramicrotome, collected on formvar/carbon-coated copper slot grids and post-contrasted with 4% uranyl acetate and lead citrate. Images were acquired on a JEM 2100 Plus transmission electron microscope (JEOL; Akishima, Japan) operated at 200 kV using a TVIPS XF 416 camera [58].

### 3.6. Real Time Proliferation Assay

Briefly, the culture medium (50 µL) was pipetted into a special E-plate with gold electrodes at the bottom of the wells and the background impedance was determined. The cell attachment and proliferation were measured as a change of impedance between electrodes and expressed as a unitless cell index. The appropriate number of cells (5000 rMSCs or 2500 C6 cells per well) was added in the culture medium (100 µL) and the cells were allowed to attach to the bottom of the wells for 2 h. This was followed by the addition of nanoparticles or their leaches in the medium (50 µL); the final particle concentration was kept at 20 µg/mL per well. The plates were then inserted into the xCELLigence RTCA DP real-time cell analyzer (ACEA Biosciences, now Agilent Technologies; Santa Clara, CA, USA) and the changes in impedance were recorded every 20 min for 3 days. All experiments were done in triplicates and repeated three times.

### 3.7. Determination of Oxidative Damage of DNA by Comet Assay

The comet assay was employed as a highly sensitive and straightforward technique for the assessment of DNA damage at the individual eukaryotic cell level. The rMSCs were prepared as described earlier and grown on the glass slides. Initially, 110 μL of normal melting point agarose, preheated to a minimum of 60 °C, was piped onto a glass slide preheated to 50 °C on a hot plate. A glass coverslip was immediately placed over the agarose, and the slide was left on ice to solidify for 5 min. The coverslip was removed and 75 μL of LMP agarose mixed with cells was added. Another glass coverslip was used to cover this layer, and it was allowed to solidify on ice for an additional 5 min. After removing the coverslip, 75 μL of pure LMP agarose was added, and the coverslip was replaced, followed by a 5 min solidification on ice. The coverslip was removed, and the specimen was gently submerged into a cooled lysing solution, taking precautions to shield it from light. It was then placed in the refrigerator for a minimum of 1 h. The samples were taken out of the lysing solution and immersed in an alkaline buffer for electrophoresis for 40 min. The electrophoresis chamber was filled with the alkaline buffer, the voltage was set at 32 V (1.2 V/cm), and a current of 300 mA was applied for 20 min at 4 °C. The specimens underwent three washes with 1 mL of 0.4 M TRIS buffer, each lasting 5 min. Following that, the samples were rinsed twice with distilled water, with each rinse lasting 5 min. The samples were immersed in 70% ethanol for 15 min and in 99% ethanol for an additional 15 min. Finally, the samples were air-dried. Lucia Comet Assay software (Laboratory Imaging; Prague, Czech Republic) was used to quantify DNA migration, expressing results as a percentage of DNA in the tail. Both total DNA damage (with enzymes) and DNA strand breaks (DNA-SB; without enzymes) were measured in 100 randomly selected cells (2 sets of 100 cells) per slide, with medians calculated from each group of 50 cells [59,60]. The level of oxidative DNA damage was assessed by comparing the median of total DNA damage with the median of DNA-SB.

### 3.8. Pilot In Vivo Imaging

As a proof of principle, the large hexagonal nanoparticles (120 nm) were applied to mice and their fluorescence was detected. For the in vivo study, the most successful candidates from in vitro investigation were selected, where large nanoparticles with Ale-PEG and Ale-(PDMA-AEA) coating were less toxic.

CD-1^®^ nude mice (Crl:CD1-FOXn1nu), 6 weeks old, were used throughout the experiments. The first experiment involved the application of nanoparticle dispersions (10 µL of UCNP@Ale-PEG or UCNP@Ale-(PDMA-AEA), concentration 4 mg/mL) subcutaneously to a mouse and scanning using the optical imager Bruker Xtreme (Bruker, Billerica, MA, USA). The excitation of the injection site was performed by a laser diode (980 nm, 100 mW) and a signal at 535 nm was detected (exposition 5 s, field of view 72 mm × 72 mm, no binning).

To evaluate the biodistribution of the nanoparticles, 100 µL of UCNP@Ale-PEG or UCNP@Ale-(PDMA-AEA) dispersion (concentration 50 µg/mL of mouse blood) was applied retroorbitally to the mice (each nanoparticle type to 5 mice). The animals were scanned at similar experimental conditions (the optical imager Bruker Xtreme, excitation using a laser diode at 980 nm, 100 mW, emission at 535 nm, exposition 1 s, field of view 190 mm × 190 mm, no binning). Due to a small area irradiated by the diode, it was not possible to excite nanoparticles in the whole animal body. Therefore, only selected organs (the liver, spleen, kidneys) were excited in several subsequent measurements. A group of two animals served as a control.

After 96 h, the mice were deeply anesthetized with chloralhydrate in a concentration of 400 mg/kg intraperitoneally and then transcardially perfused first with phosphate buffer (PB) and then with 4% paraformaldehyde in PB. Paraformaldehyde-fixed tissue samples (kidney and liver) were cryopreserved in sucrose solution with gradually increasing concentration (10, 20, and 30% sucrose in deionized water). The tissue was then embedded in OCT mounting media (VWR; Radnor, USA). Sections were cut on a Cryostar NX70 cryostat (Thermo Fisher Scientific; Waltham, MA, USA) to 30 µm sections (liver and kidney) and stained with DAPI for 10 min. Finally, the content of Yb and Y was quantified by inductively coupled plasma mass spectrometry (ICP-MS).

The animal experiments were performed in accordance with national and international guidelines for laboratory animal care and were approved by the Laboratory Animal Care and Use Committee of the First Faculty of Medicine, Charles University, and the Ministry of Education, Youth and Sports of the Czech Republic (MSMT 46304/2020-3).

### 3.9. Laser Scanning Confocal Microscopy

To visualize the UCNPs, a Carl Zeiss LSM 880 NLO microscope (Oberkochen, Germany), equipped with a 40× NA1.1 water immersion objective and a 32 GaAsP array spectral detector covering emission from 410 to 694 nm and operated at single photon counting mode for maximum SNR, was used. Lambda mode at full spectral resolution was used to spectrally prove UCNPs emission (two characteristic distinct and narrow peaks at 544 nm and 660 nm) and a channel mode was used to combine DAPI (405 nm excitation, 410–500 nm emission bands), UCNPs (974 nm excitation, 535–565 nm and 650–668 nm emission bands), and transmitted light signals. A 974 nm excitation by a TiSa laser (80 MHz, 350 fs laser pulse width at sample plane) provided the highest emission intensity for upconversion and was used for imaging. The 974 nm laser power was kept low at <50 µW at the sample plane. To capture the slow emission of UCNPs (excited state lifetime on the order of hundreds of microseconds), the scanning speed was the slowest possible, 132 µs per pixel, and the pinhole was opened to 300 um (around 4 Airy units). The pixel size was 132 nm, the image size was 512 × 512 pixels, and bidirectional scanning was used.

### 3.10. Inductively Coupled Plasma Mass Spectrometry (ICP-MS)

A NexION 350D ICP-MS instrument (PerkinElmer; Shelton, WA, USA) equipped with Universal Cell Technology™ for spectral interference elimination was used for ICP-MS measurement. The sample introduction system included an internal peristaltic pump with Tygon^®^ tubing (0.38 mm internal diameter), a polytetrafluorethylene concentric nebulizer, and a glass cyclonic spray chamber with a volume of 100 mL. For the measurement of 89 Y and 174 Yb isotopes, the samples were diluted with 2% nitric acid and spiked with the internal standard (IS) solution (103 Rh).

Calibrated Y and Yb solutions and IS solution were prepared from solutions of concentration 1.000 ± 0.002 g/L (Merck, Darmstad, Deutschland).

### 3.11. Statistical Methods

The data are presented as the mean ± standard deviation (SD). Statistical analyses were conducted using the GraphPad Software (GraphPad Prism, version 9), employing a one-way ANOVA test followed by Tukey’s post hoc test. For evaluation of the 72 h cytotoxicity, we used one-way ANOVA with multiple comparisons vs. control, and the Bonferroni *t*-test. A significance threshold of *p* < 0.05 was applied to determine statistical significance.

## 4. Conclusions

In this report, the long-term cyto- and genotoxicity of the particles and/or their extracts was evaluated to better understand the hazards associated with the biological application of UCNPs. In addition, some important information on the biodistribution, clearance, and accumulation of particles in tissue organs in vivo was obtained. Both large (120 nm according to TEM) and small (25 nm) UCNPs can be internalized in the cell cytoplasm. A real-time proliferation assay has confirmed that L-UCNPs were less toxic than the S-UCNPs. The presence of Y and Yb in the leachates confirmed the higher solubility of S-UCNPs in the medium than L-UCNPs, which corresponded to the higher cytotoxicity of the leachates from small nanoparticles, especially in C6 cells. The coating with PMVEMA did not provide sufficient protection against toxicity after incubation with the cells. On the contrary, S-UCNPs caused very little oxidative damage; the significantly higher oxidative damage was found after neat L-UCNPs treatment in rMSCs and was diminished after coating with Ale-(PDMA-AEA), PMVEMA, and Ale-PEG. The C6 cell line was less sensitive to oxidative damage than the primary culture (rMSCs). An in vivo study showed that both types of nanoparticles were eliminated from the body via the liver. While L-UCNPs@Ale-PEG particles were almost completely eliminated from the liver 96 h after intravenous application, L-UCNPs@Ale-(PDMA-AEA) particles remained in the liver in a significantly higher amount. The polymeric coating can influence the retention of nanoparticles in tissues to some extent. This can be advantageously used in various applications using cell labeling in tissues and organs. In conclusion, the study’s findings highlight the promising translational potential of surface-engineered upconversion nanoparticles (UCNPs) in nanomedicine. However, comprehensive preclinical studies are imperative to assess the biodistribution, pharmacokinetics, and long-term safety profiles of functionalized UCNPs, including evaluations of acute and chronic toxicity, immune responses, and potential off-target effects in animal models. Furthermore, additional investigations into the immunogenicity, biodegradation, and efficacy of UCNPs-based formulations in disease-specific animal models are necessary to support their clinical translation, with a focus on optimizing delivery strategies and enhancing therapeutic outcomes. Iterative design and testing of UCNPs-based formulations, surface coatings, and targeting ligands are essential to improve their pharmacokinetic properties, specificity, and biocompatibility for clinical applications, ensuring their safety and efficacy in diverse patient populations while addressing the remaining challenges in nanomedicine.

## Figures and Tables

**Table 1:** Characterization of neat and polymer-coated UCNPs.

	S-UCNPs				L-UCNPs			
Coating	*D*_n_ (nm)	*Đ*	*D*_h_ (nm)	ζ-Potential (mV)	*D*_n_ (nm)	*Đ*	*D*_h_ (nm)	ζ-Potential (mV)
-	25	1.01	101	30 ± 5	121	1.01	174	28 ± 3
Ale-PEG	25	1.01	68	8 ± 1	119	1.02	158	4 ± 1
Ale-(PDMA-AEA)	25	1.01	102	27 ± 3	122	1.01	160	22 ± 2
PMVEMA	25	1.01	112	−32 ± 2	119	1.01	234	−46 ± 6

UCNPs—upconverting NaYF_4_:Yb^3+^,Er^3+^ nanoparticles; Ale-PEG—poly(ethylene glycol)-alendronate; Ale-(PDMA-AEA)—poly(*N*,*N*-dimethylacrylamide-*co*-2-aminoethylacrylamide)-alendronate; PMVEMA—poly(methyl vinyl ether-*co*-maleic acid); *D*_n_—number-average diameter (TEM); *Ð*—dispersity (*D*_w_/*D*_n_; TEM); *D*_h_—hydrodynamic diameter (DLS).

## Data Availability

The data will be published at Zenodo repository.
